# Perception of pointing gestures in 3D space

**DOI:** 10.1038/s41598-024-78129-4

**Published:** 2024-11-11

**Authors:** Lisa-Marie Krause, Oliver Herbort

**Affiliations:** Department of Psychology, Julius-Maximilians-Universität Würzburg, Röntgenring 11, 97070 Würzburg, Germany

**Keywords:** Pointing gestures, Visual perception, Deixis, Virtual reality, Non-verbal communication, Psychology, Human behaviour

## Abstract

Pointing gestures are often used to refer to distant referents by indicating in which vertical and horizontal direction the referent is located relative to the pointer. In the present manuscript, we address whether and how both dimensions interact when people spatially interpret pointing gestures, or whether both dimensions are processed independently as reflected in many current models. We found that both dimensions interact on different levels. First, cross-dimensional effects were found on a between-gestures level. That is, the perception of the vertical position implied by a pointing gesture depended on horizontal arm and finger orientation. Conversely, the horizontal interpretation depended on vertical arm and finger orientation. Second, we found cross-dimensional interactions on the level of intra-individual biases. That is, participants’ horizontal perceptual biases in interpretations (e.g., perceiving a gesture as directed more rightward than others) were related to their vertical perceptual biases. Third, we found cross-dimensional interactions on the level of intra-individual variability. That is, the vertical and horizontal interpretations of the same pointing gestures were correlated within participants and gestures. Together, these findings indicate that human spatial pointing perception is based on configural processing of a gesture on different levels of information processing.

## Introduction

Pointing gestures are an important building block of our daily communication. People point when they aim to direct the focus of others to objects, persons, or events of interest. In other words, when they aim to achieve joint attention^1^. Pointing gestures may be complemented by verbal descriptions, but there are also situations where those are hardly possible and thus pointing carries the bulk of the information. Examples are when pointing at objects that lack salience such as a star in the night sky, or when pointing towards a small, well-camouflaged animal in its natural habitat. Producing and interpreting pointing gestures in their various appearances like pointing with or without additional verbal information, pointing with different body parts like head, nose, or hand and index finger are subjects of discussion for some decades now^2–6^. Although the exact execution of a manual pointing gesture depends on the situation and the intention of the pointer, most often he will extend his (right) arm, hand, and index finger in a straight line towards the target. Straight pointing gestures are not only easier to produce and thus, less error prone in production, but also lead to higher readability that diminishes the risk of misinterpretations compared to bent gestures with more degrees of freedom^7,8^. In the following, we solely focus on straight points.

In the mentioned examples and many other situations, pointing gestures convey information about the horizontal and vertical direction in which a referent can be found (e.g., a star or a nuthatch). This raises the question of how observers perceive, process, and interpret information concerning both dimensions. Two opposite hypotheses can be distinguished. First, the horizontal and vertical components of a pointing gesture could be perceived as a set of independent elements. In this *elemental* approach, the elevation (vertical orientation) and azimuth (horizontal orientation) of the pointing arm and finger are separately included into the final percept. Accordingly, no interaction between dimensions should occur and the interpretation of the vertical gesture orientation should not be affected by its horizontal orientation, and vice versa. This assumption is implicit in approaches that treat human pointing interpretation as some form of vector extrapolation^6,9–11^ and supported by the accuracy of such models. Likewise, methods that build on vector models but include corrections to more closely match empirical data often treat both dimensions independently, further closing the gap between models using the elemental approach and empirical data^12^.

Second, observers could perceive the whole pointing gesture as a *configuration*. According to the configural approach, all information is processed together and therefore, the judgment for one nominal dimension could interact with the other. These considerations are supported by the first experiment of Mayer et al.^12^ who examined biases in pointing interpretation in a virtual reality setting—to develop the above-mentioned correction model. Visual inspection of their data indicated a slight interaction between the vertical and horizontal dimension. From some observer viewpoints, upward points were descriptively perceived more to the left compared to downward ones although the arm azimuth remained the same. In another experiment, Krause and Herbort^9^ manipulated a pointer’s horizontal arm orientation and found that it affected vertical pointer-observer misunderstandings as well. Importantly, these differences in misunderstandings can be attributed to effects in the observers’ interpretations. In a third experiment, in which the elevation and azimuth of a pointer’s arm was manipulated, horizontal judgments slightly depended on arm elevation^9^.

In summary, previous results do not exclusively favor one over the other approach and therefore, do not allow for a definite conclusion at this stage. On the one hand, models using the elemental approach provide a good approximation to the empirical data. On the other hand, a more detailed look at the data suggests interactions between dimensions. Especially, the effect of arm azimuth on vertical interpretations has been documented. However, such interactions have rarely been in the focus of systematic research so far. Thus, in this paper, we systematically address whether human interpretations of pointing gestures can be best described by an elemental approach—thus are derived independently for each dimension—or whether both dimensions interact as predicted by the configural approach.

**Fig. 1:**
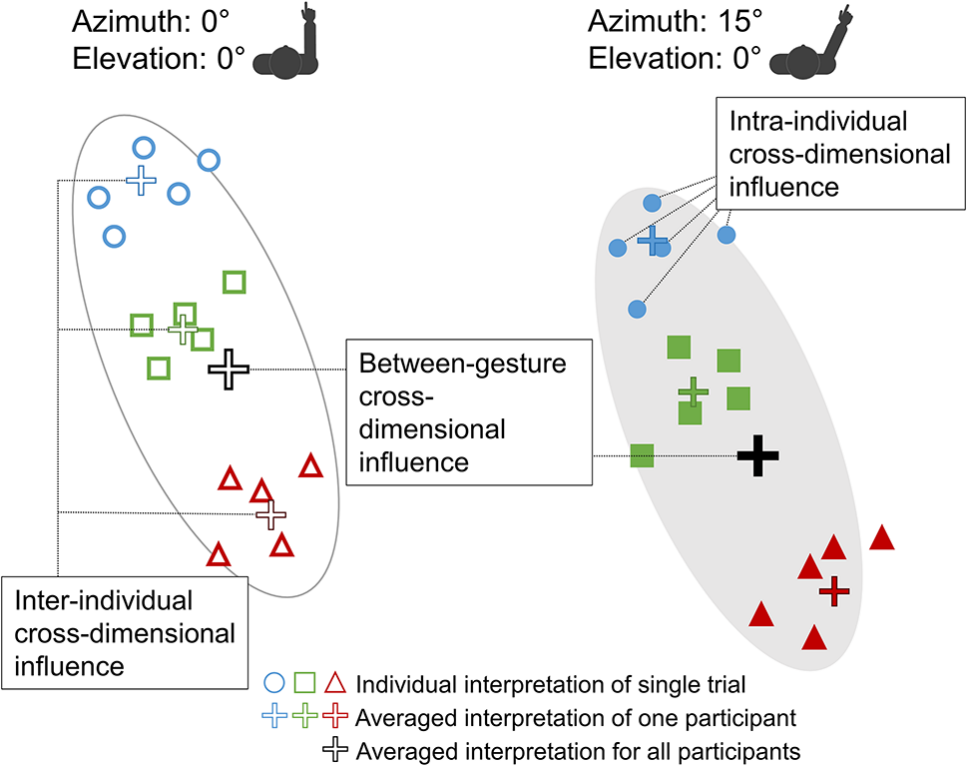
Overview of different levels of cross-dimensional influence. Illustration of between-gestures, inter-individual and intra-individual levels of cross-dimensional influence. Interpretation pattern of two pointing gestures with same elevation but different azimuths (left/right ellipse) by three different observers (blue circle, green square, red triangle).

Cross-dimensional interactions could become evident on three different levels: on the between-gestures level, on the level of inter-individual perceptual biases, and on the level of intra-individual variability resulting from same gesture. Figure 1 illustrates the three examined levels. First, both dimensions could interact on the *between-gestures level*. This level is concerned with the population’s average interpretation of different pointing gestures (black plus symbols in Fig. 1). If both dimensions interact, the vertical interpretation of a pointing gesture would depend on the arm azimuth. Likewise, the horizontal interpretation could depend on the arm elevation. In practice, considering interactions on this level would allow for an improvement of geometric pointing models that make predictions about the average interpretation of the average person. These models could be used, for example, to generate pointing gestures of robots or virtual characters^13^ or correct those of avatars^11,14^ to improve their understandability for the average person.

Second, *inter-individual variability on the within-gesture level* deals with different “perceptual styles” of observers. People show inter-individual variability in visual perception including orientation perception^15–17^. Therefore, participants might differ in how they process pointing gestures on average. For example, one person might perceive a specific gesture as indicating a more rightward and more upward position on average than another person (e.g. colored and filled plus symbols in Fig. 1). If both dimensions interact participants’ horizontal and vertical biases would be correlated for each gesture. Therefore, the analysis provides information about the individual peculiarities in gesture perception. In practice, the results would allow, for example, to more easily tailor pointing gestures or their corrections to individual users by considering the structure of their respective perceptual biases.

Third, *intra-individual variability on the within-gesture level* refers to an observer’s repeated interpretation of the vertical and horizontal component of a specific pointing gesture. If pointing gestures are processed as a configuration on this level, variability in both dimensions is expected to be correlated (shapes of the same color and fill in Fig. 1). For example, a relatively rightward interpretation of a specific gesture might also be more likely to be relatively upward (or downward). Thus, analyses at this level describe the structure of individual variability of an observer in response to the same stimulus. In practice, information about this type of interaction could be used to generate pointing gestures that minimize ambiguity, for example by maximizing the overlap between a referent and the distribution of possible interpretations of a specific gesture.

### Current study

We conducted an experiment to address the effect of cross-dimensional interactions when interpreting pointing gestures. For this reason, we asked participants to interpret different pointing gestures of a virtual, pre-programmed pointer. Pointing gestures were produced by orthogonally manipulated arm azimuth and arm elevation and were directed towards a wall in front of the pointer.

We aimed to investigate the interplay between the vertical and horizontal dimension within interpretations of pointing gestures in a 3-dimensional design by analyzing averaged patterns across participants (between-gestures level). In addition, we also focus on inter- and intra-individual characteristics in the perception of a single pointing gesture. Information about such relationships would be important to understand the processes involved in pointing perception and would be a critical step towards various real-life applications. To our knowledge, these effects have not yet been analyzed so far.

Furthermore, we also aim to further examine another factor that affects pointing interpretation—the distance between pointer and referent^18^. It is already known that observers do not extrapolate the pointing arm linearly over distance when being presented the side-view of a pointer on a 2D screen, leading to interpretations biased towards the pointer’s shoulder height^6^. In contrast, we still do not know, whether and how this non-linearity affects interpretations in 3D. Thus, we additionally used three different distances between pointer and the wall on which was pointed.

To acknowledge the great situational variety in which pointing gestures are used in, and thus, to allow for a broader generalization of results, we lastly integrated two different observer viewpoints, one behind the pointer and one to his side. We chose these specific viewpoints for the following reasons. First, we consider that similar viewpoints are held frequently in every-day pointing-based communication. For example, when only two people are involved, the observer may intuitively step behind the pointer to get a better reading of the gesture. In larger groups, such as guided tours, observers are more likely to see the pointer at some distance and from the side. Second, the interpretation of pointing gestures can be generally understood as the combination of two distinct visual cues^9^. The first visual cue refers to the position of whichever object the observer sees behind the pointer’s finger (position cue). The second visual cue refers to the position that results from extrapolating the pointing arm and finger (direction cue). It has been shown that while the direction cue is the primary determinant of interpretations when pointing gestures are seen from the side, the relative influence of the position increase, when the observer approaches position closer to the pointer^9,19^. Thus, as pointing interpretations can be understood as the combination of a direction and position cue, we choose to present viewpoints in which either cue can be expected to be the primary determinant of interpretation. We expect that this covers the central processes involved in the spatial interpretation of pointing and facilitates the generalizability of our findings. Third, we used viewpoints resembling those used in previous studies^9^, allowing us to build upon and connect our results to previous research. Note that the different viewpoints differ on a multitude of dimensions (e.g., distance to pointer, relative position to pointed-at surface) and we thus expect that the observer perspective has a considerable effect on interpretations. However, we do not aim to examine this effect in the present manuscript. Rather, we include two different viewpoints to increase the generalizability of any results. Hence, we treat data of both observer viewpoints independently to simplify the analysis. The effect of observer perspective has been treated in more detail elsewhere^6,9,12^.

Taken together, our primary objectives are the following. First, the considerations outlined above suggest a clear effect of arm azimuth on vertical judgments on the between-gestures level, which we also expect to find in our experiment. We additionally want to test whether arm elevation in turn affects horizontal judgments. Second, we want to examine whether vertical and horizontal information of a pointing gesture interact on both within-gesture levels. If gesture interpretation is following the configurational approach, we expect to find significant correlations between vertical and horizontal interpretations in a predominant proportion of pointing gestures—within the repeated interpretations of a single observer as well as between different observers’ average interpretations. If the elemental approach describes pointing interpretation best, we generally do not expect significant correlations between vertical and horizontal interpretations—again within and between observers. Third, interpretations of pointing gestures have been shown to be nonlinear. When seen from side, vertical arm extrapolation are relatively linear for short pointer-referent distances but gravitate toward a horizontal axis if the distance increases. Such nonlinearities have not been examined in a three-dimensional setup so far. Moreover, we want to examine whether such non-linearity biases also exist for horizontal judgments.

## Methods

### Participants

23 volunteers (eight males) recruited online from the participant pool of the Department of Psychology of the University of Würzburg between 20 and 56 years (*M* = 32.9) completed the experiment. Except for one participant, all were right-handed. All gave written informed consent and were compensated with money for voluntary participation. This experiment was approved by the ethics committee of the Department of Psychology of the University of Würzburg (GZEK 2019-20) and conducted in accordance with the Declaration of Helsinki.

For this study, we aimed at a power of 80% for detecting cross-dimensional effects. That is, first, an effect of pointer’s arm elevation on horizontal judgments that corresponded to 10% of the effect of arm azimuth. Second, an effect of arm azimuth on vertical judgments again corresponding to 10% of the effect of arm elevation. Six participants conducted the experiment beforehand to generate pilot data (*n* = 6). Based on this data, we repeatedly generated two data sets to model the effect of arm elevation on horizontal judgments and vice versa. To generate a horizontal data set, we replaced the horizontal judgements with the mean horizontal judgements of the respective combination of the factors observer viewpoint, distance, and arm azimuth and then added the expected effect of the arm elevation. In the pilot data, a change of the arm azimuth by 1° resulted in a change of the horizontal judgment by about 0.83°. Hence, the expected effect was determined by multiplying the arm elevation with 0.083°. Data for hypothetical participants were generated by multiplying this data set and adding normal distributed noise based on the intra-individual and intra-condition standard deviations obtained from the pilot. Each data set was subjected to a repeated measures ANOVA with within factors arm azimuth, arm elevation, distance, and observer viewpoint, including the Bonferroni–Holm correction. When the ANOVA revealed a significant effect of arm elevation or a significant interaction involving arm elevation, the effect of arm elevation was considered detected. To estimate the power, the relative frequency of simulations in which an effect was detected was computed. This procedure was conducted analogously for the effect of the arm azimuths on vertical judgements, except that the effect was computed by multiplying the arm azimuth with 0.074°. Using a custom R script, we simulated 512 experiments for various sample size (4, 8, 16, 20, 24, 32). The simulations showed that a sample size of *n* = 20 allowed to detect effects of arm elevation on horizontal judgments with a power of 1 − *β* = 0.83 and that a sample size of *n* = 4 allows the detection of an effect of arm azimuth on vertical judgments with a power of 1 − *β* = 0.88. Based on these considerations, we scheduled 12 two-participant sessions to collect valid data of at least the calculated sample size of *n* = 20.

### Stimuli and apparatus

**Fig. 2:**
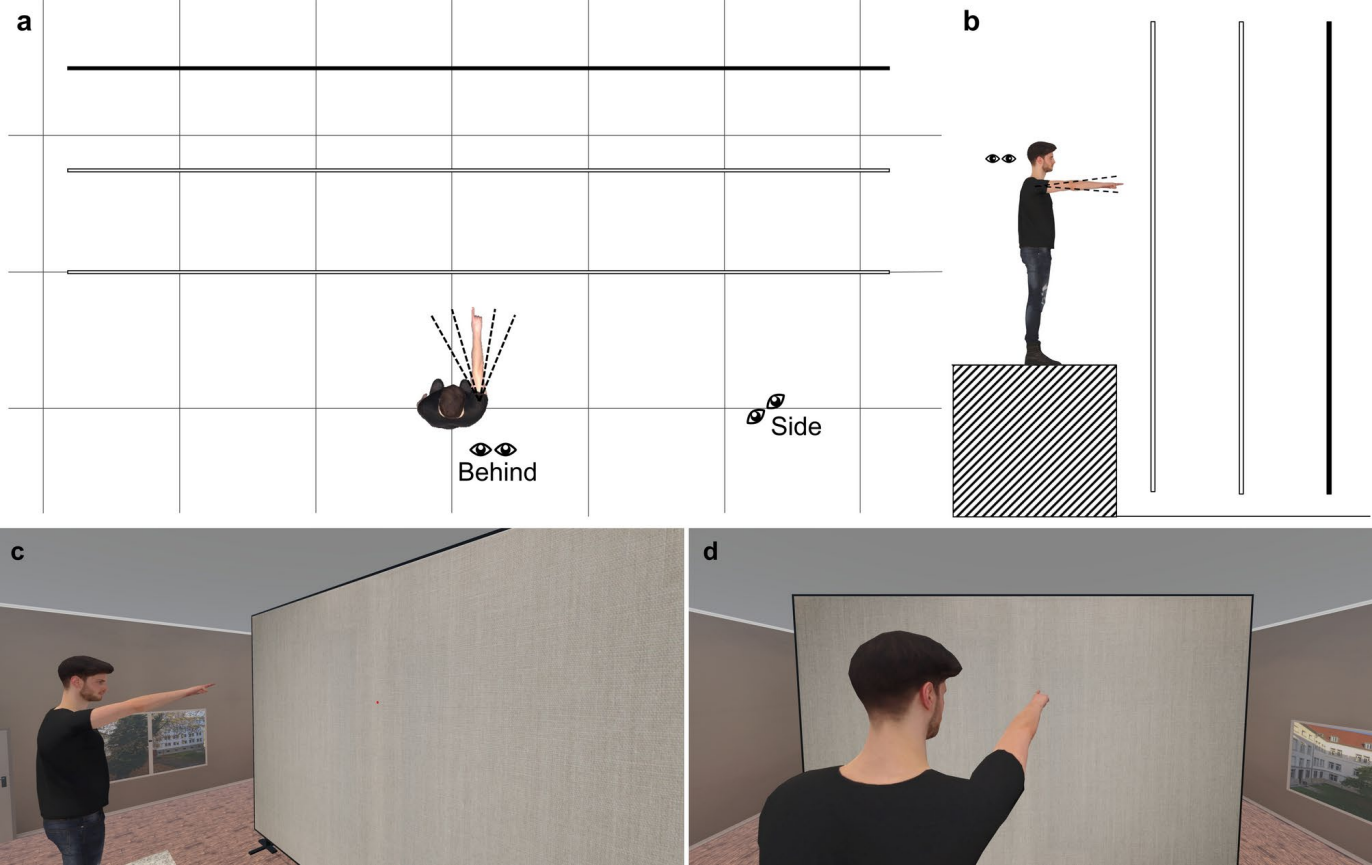
Spatial layout of the experimental design. Experimental setup with the pointing gestures targeting the wall. Black, solid lines represent the wall at its three possible distances (100 cm, 175 cm, 250 cm). (**a**) Layout from bird’s-eye-perspective. The pointer’s arm marks the azimuth = 0°. The other implemented azimuths to the left and right side are plotted as dashed lines. Eyes indicate both observer viewpoints. Light gray squares of the grid are 100 cm × 100 cm. (**b**) Observer’s eye level is represented by the pair of eyes. The pointer’s arm marks the elevation = 0°. Other implemented elevations are indicated by black dashed lines. (**c**) Observer perspective from side viewpoint (azimuth = 12.5°, elevation = 15°, distance = 175 cm). (**d**) Observer perspective from behind viewpoint (azimuth = 12.5°, elevation = 15°, distance = 175 cm).

Participants sat in front of a small desk, put on an HTC Vive Pro or Pro Eye head-mounted display (HMD), and used a wireless mouse with their dominant hand as input device. Both HMDs had equal display specifications. The eye tracking function of the Pro Eye was not used. Participants always held the observer role. Figure 2 depicts the experimental layout as well as observer’s perspective from both viewpoints. A male virtual agent served as pointer (height: 1.92 m; shoulder-to-fingertip-distance: 0.76 m) and stood on a 1.3 m high platform in a room. The pointer faced a wall (6 m × 4 m) painted with slightly structured wallpaper to strengthen depth perception. The usage of the pedestal and the quite large wall ensured that also downward points were directed on the wall and additionally, that enough buffer space were around all pointed-at locations so that observers perceived all points from both viewpoints as directed on the wall. A red circle with a white dot in its center served the participants as cursor, which could be moved on the wall with a computer mouse. Depending on condition, participants observed the scene either from 30 cm behind the pointer’s right shoulder or from a viewpoint 2.3 m next to his right shoulder^9^. These specific viewpoint locations guaranteed that the fingertip was clearly visible at the behind viewpoint for all preprogrammed gestures and that the whole wall was in view for the side viewpoint. Viewpoint height was fixed for all participants at 175 cm. To avoid simulator sickness, participants could slightly move their heads but as soon as deviating more than 15 cm in any direction from the defined viewpoint, their virtual head position was reset to the initial position. Coordinate system of the VR environment was rotated so that each trial starts with the pointing finger in front of the observer when the participant’s head was oriented forward in real world.

Pointing gestures were always presented as right arm, hand, and index finger of the static pointer being outstretched in a straight line. At the same time, the virtual pointer was looking at an imaginary point on the wall that resulted from extrapolating a vector from his cyclopean eye over index fingertip, thus mimicking gaze behavior during natural pointing^2,9^.

### Procedure

At each trial onset, the pointer pseudo-randomly performed one of 15 preprogrammed pointing gestures to hypothetical, invisible referents on the wall. For generating these gestures, arm azimuth was varied between angles from −25° (far left) over −12.5°, 0° (straight ahead), 12.5° to 25° (far right). Additionally, three different arm elevations were used, 15° (upward), 0° (parallel to the floor), and −15° (downward). Simultaneously, the cursor appeared in the center of the wall. After moving it with the mouse to the estimated referent position, participants confirm their choice by pressing the left mouse button. Subsequently, the cursor disappeared, and the pointer was shown with a lowered arm and straight head orientation for 500 ms before the next trial started.

The experiment consisted of 12 blocks, which differed with respect to observers’ viewpoint (behind the pointer, to his right side) and distance between wall and pointer (100 cm, 175 cm, 250 cm), and were presented in pseudo-random order. All 15 gestures were presented five times in each block. Trial order was pseudo-randomized. Each block type was presented twice and after each block, participants had the option of a short, self-paced break. In sum, the experiment comprised 900 trials and took approximately 35 min.

### Dependent variable and data reduction

**Fig. 3:**
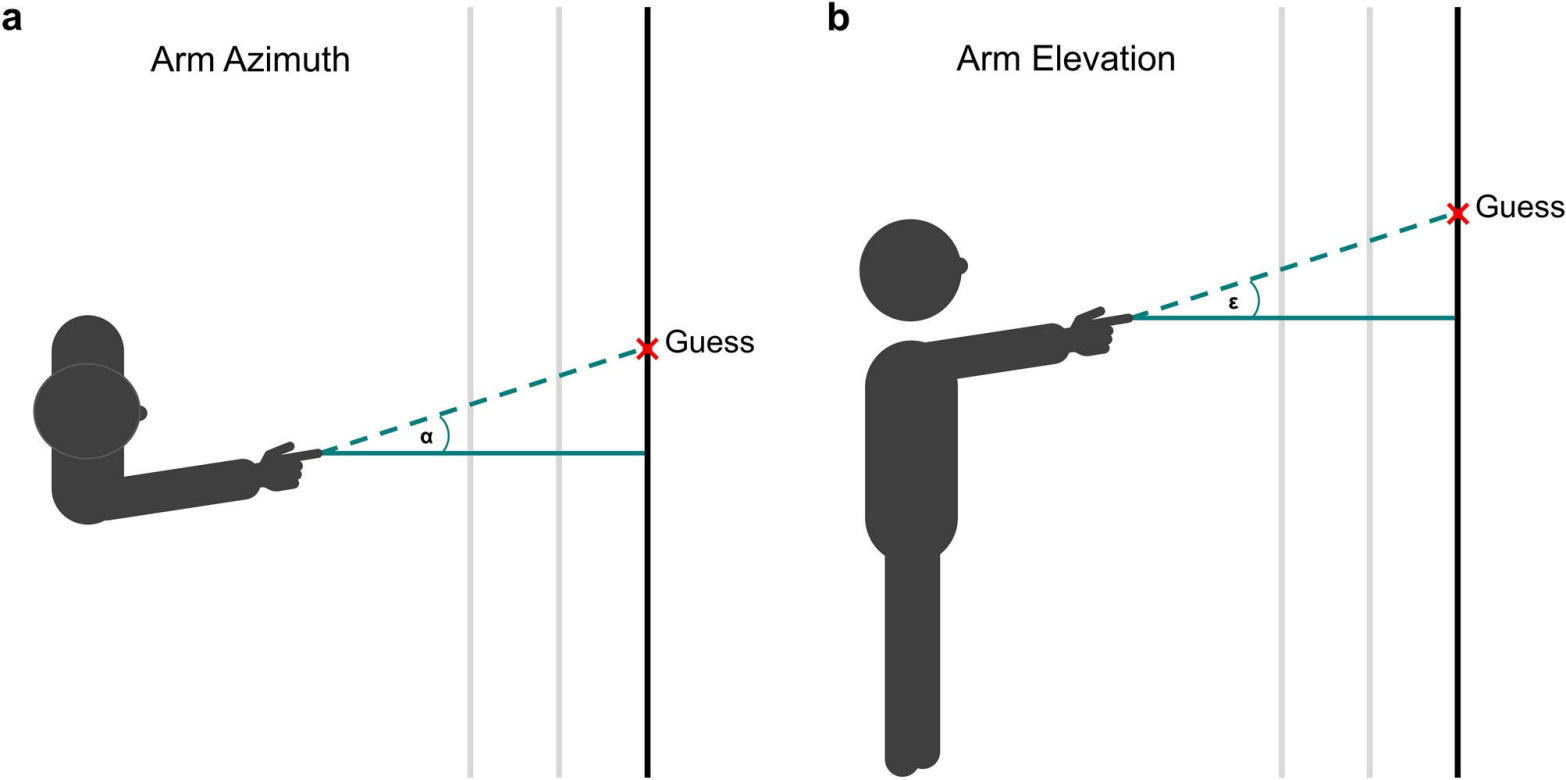
Graphical representation of dependent variables. Calculation of horizontal (**a**) and vertical (**b**) angle between participant’s guess and finger line. Vertical bars represent different wall distances. When observers linearly extrapolate arm/finger direction, these angles are of the same size as arm azimuth (**a**) and arm elevation (**b**), respectively.

As pointing interpretations are starting at pointer’s fingertip^9^, we decided to calculate the vertical and horizontal angle between the line from pointer’s fingertip to participant’s guess and sagittal axis as dependent variable (Fig. 3). Positive values indicate that participant perceived arm and finger further right- and upwards. This operationalization has the advantage that interpretations are directly comparable to gesture’s azimuth and elevation since deviating angles indicate biased gesture extrapolations. Furthermore, non-linear judgments over distance are directly visible if an effect of distance shows up since any non-linear extrapolations would result in different angles for each distance.

Participants completed 12 warm-up trials, which were omitted from analysis. As the data of one participant was not recorded completely, he was excluded from further analysis, resulting in 22 remaining participants. Two participants reported afterwards difficulties with depth perception and/or spatial vision in everyday life. However, a visual inspection of their data revealed that they were descriptively similar—with respect to point arrangement for all cells—to the data of other participants. Hence, these participants remained. As outlier criterion, we calculated *z*-standardized residuals for both dimensions of each interpretation (data split by participant, arm azimuth, arm elevation, observer viewpoint, and distance to wall) and omitted all trials that were more extreme than [−2|2] on at least one dimension. This resulted in an exclusion of 8.3% of all remaining trials.

## Results

To analyze potential cross-dimensional effects on different levels in pointing interpretation, we structured the results section from a macroscopic to a microscopic perspective. That is, we start with broader analysis of how observers generally interpret pointing gestures on both dimensions and look for between-gestures variability. Then, we especially focus on the single gesture (within-gesture levels) to find potential correlations between both dimensions. First, for interpretations of different observers (inter-individual level) and finally, for interpretations of a single observer (intra-individual level).

### Cross-dimensional effects between gestures

**Fig. 4:**
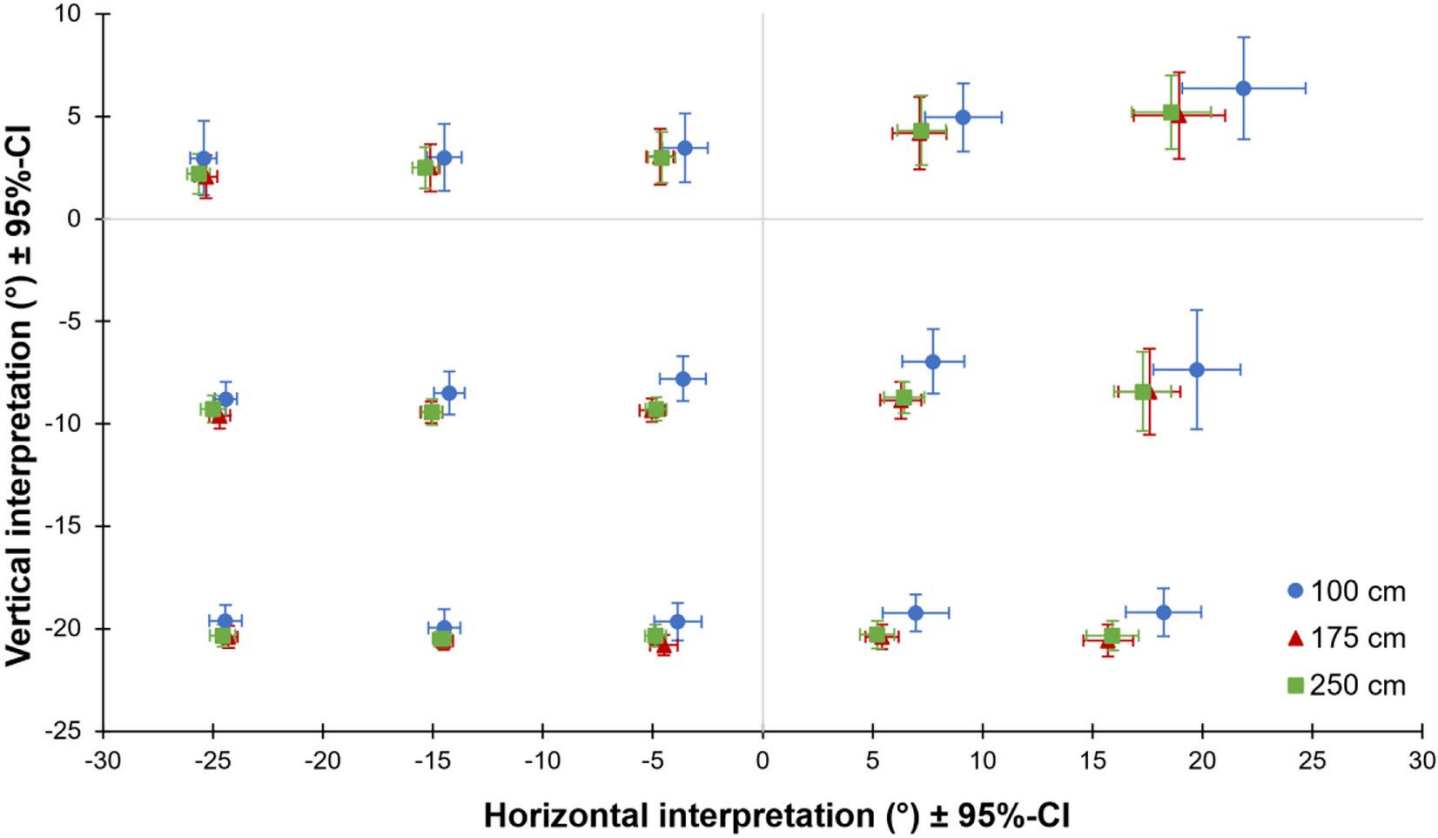
Interpretations from behind viewpoint. The figure shows observers’ vertical and horizontal pointing interpretation from behind pointer’s right shoulder for each of 15 pointing gestures and all three different distances between pointer and wall. Error bars show 95% confidence intervals (CI).

**Fig. 5:**
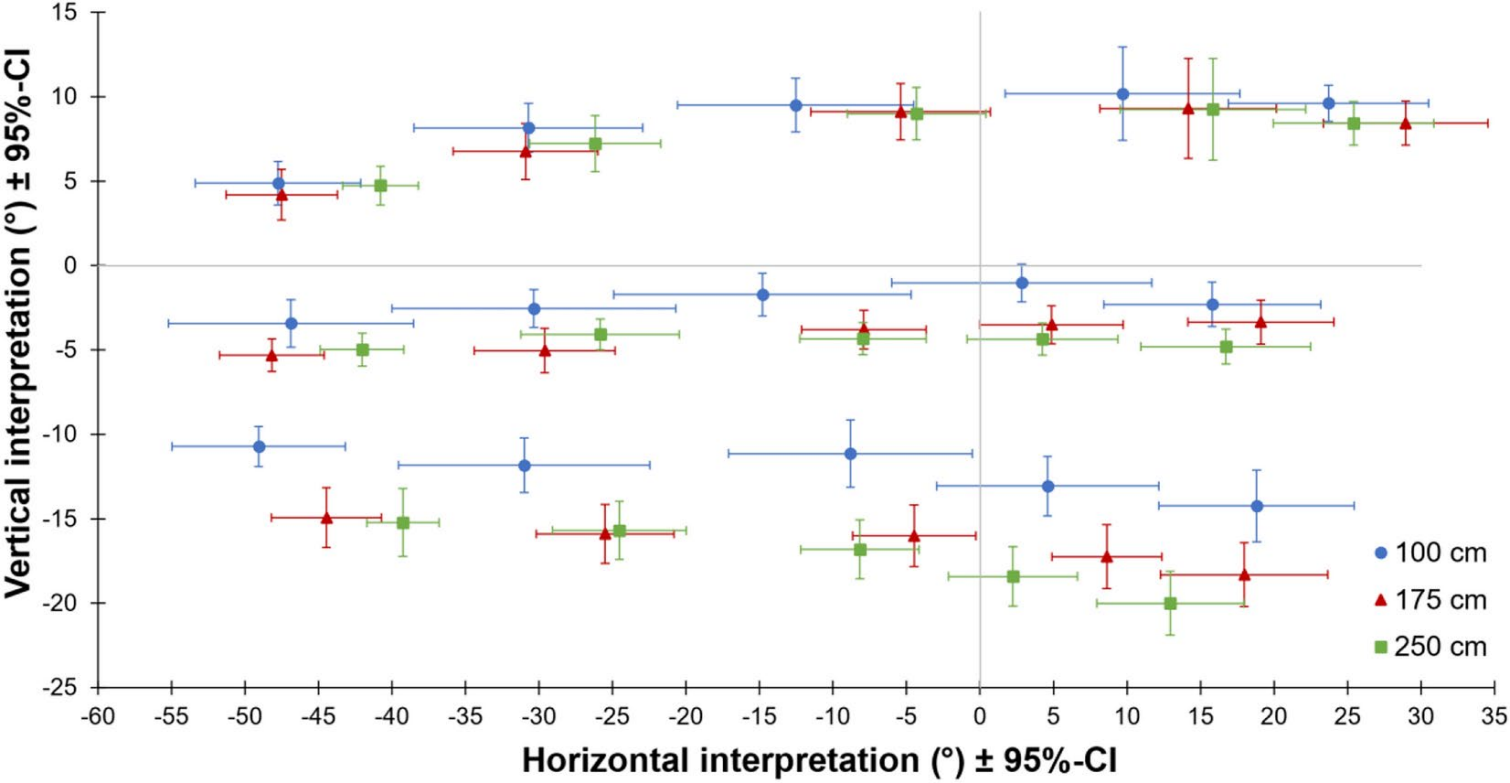
Interpretations from side viewpoint. The figure shows observers’ vertical and horizontal pointing interpretation from pointer’s right side for each of 15 pointing gestures and all three different distances between pointer and wall. Error bars show 95% confidence intervals (CI).

The effect of arm azimuth on vertical interpretations of pointing gestures and of arm elevation on horizontal interpretations was analyzed for each observer viewpoint and dimension separated with repeated measures ANOVAs with within-subject factors arm azimuth (−25°, −12.5°, 0°, 12.5°, 25°), arm elevation (15°, 0°, −15°) and distance between pointer and wall (100 cm, 175 cm, 250 cm). Observer interpretations for both observer viewpoints in dependence to distance are shown in Fig. 4 (behind viewpoint) and Fig. 5 (side viewpoint). Note that since we do not aim for comparing cross-dimensional effects between both observer viewpoints but rather for a broader generalizability of the findings for different situations, observer viewpoints were analyzed separately to keep the analyses and interpretation straight and simpler. For the sake of completeness, the results of the ANOVAs additionally including observer viewpoint as factor can be found in Supplemental Table 1 (horizontal judgments) and Supplementary Table 2 (vertical judgments). The results of the ANOVAs, including Greenhouse–Geisser corrected *p*-values, are reported in Table 1. All *p*-values survived Bonferroni-Holm correction for multiple tests of each ANOVA. Only significant main effects and interactions are reported below, starting with those concerning a cross-dimensional influence, followed by those concerning linearity. The obvious and strong effects of arm azimuth on horizontal interpretations and arm elevation on vertical interpretations are not discussed.

**Table 1:** Results of ANOVA for both viewpoints and both dimensions.

Effect	Behind viewpoint					Side viewpoint				
	*F*	*df*	*p*	${\eta }_{p}^{2}$	*ε*	*F*	*df*	*p*	${\eta }_{p}^{2}$	*ε*
Horizontal dimension										
Arm Azimuth	**2525.84**	**4,84**	** < 0.001**	**0.99**	**0.29**	**392.54**	**4,84**	** < 0.001**	**0.95**	**0.37**
Arm elevation	**6.26**	**2,42**	**0.015**	**0.23**	**0.60**	**11.41**	**2,42**	** < 0.001**	**0.35**	**0.91**
Distance	**10.28**	**2,42**	** < 0.001**	**0.33**	**0.76**	0.98	2,42	0.339	0.05	0.54
Azimuth × elevation	**21.96**	**8,168**	** < 0.001**	**0.51**	**0.28**	**18.36**	**8,168**	** < 0.001**	**0.47**	**0.63**
Azimuth × distance	**8.88**	**8,168**	** < 0.001**	**0.30**	**0.34**	**5.71**	**8,168**	**0.003**	**0.21**	**0.33**
Elevation × distance	0.78	4,84	0.477	0.04	0.55	**4.00**	**4,84**	**0.012**	**0.16**	**0.74**
Azimuth × elevation × distance	1.03	16,336	0.412	0.05	0.38	**3.54**	**16,336**	**0.002**	**0.14**	**0.42**
Vertical dimension										
Arm Azimuth	**16.35**	**4,84**	** < 0.001**	**0.44**	**0.32**	**14.14**	**4,84**	** < 0.001**	**0.40**	**0.63**
Arm elevation	**1567.09**	**2,42**	** < 0.001**	**0.99**	**0.52**	**910.47**	**2,42**	** < 0.001**	**0.98**	**0.57**
Distance	**11.20**	**2,42**	** < 0.001**	**0.35**	**0.73**	**20.70**	**2,42**	** < 0.001**	**0.50**	**0.79**
Azimuth × elevation	**16.77**	**8,168**	** < 0.001**	**0.44**	**0.53**	**57.85**	**8,168**	** < 0.001**	**0.73**	**0.56**
Azimuth × distance	1.17	8**,**168	0.330	0.05	0.51	**3.87**	**8,168**	**0.002**	**0.16**	**0.70**
Elevation × distance	1.06	4**,**84	0.371	0.05	0.69	**26.07**	**4,84**	** < 0.001**	**0.55**	**0.62**
Azimuth × elevation × distance	1.05	16**,**336	0.397	0.05	0.43	1.32	16**,**336	0.247	0.06	0.42

Significant main effects and interactions are printed in bold.

### Horizontal interpretation from behind viewpoint

Downward points were interpreted more to the left than upward points. Elevation affected horizontal interpretations of right points but had only little to no influence when pointing to the left or straight ahead. Thus, arm elevation affected mean horizontal interpretations. Points to the 100 cm wall were perceived as more to the right than to both other distances. At the 100 cm distance, but not at the other distances, interpretations were further to the right, the further the arm moved from left to right. Thus, the horizontal component of interpretations depended non-linearly on distance.

### Horizontal interpretation from side viewpoint

Points parallel to the floor were interpreted more leftward compared to upward or downward ones. An upward gesture led to stronger rightward interpretations when pointing to the right than when pointing straight ahead or to the left. Downward points at 100 cm and 175 cm were perceived more rightward than points parallel to the floor. In contrast, pointing downward at 250 cm led to minimal more leftward interpretations than for other arm elevations. In summary, arm elevation again affected mean horizontal interpretations. Leftward pointing gestures were descriptively perceived most rightward at 250 cm while for all other azimuth levels the interpretation fell most rightward at 175 cm. Thus, arm elevation again affected mean horizontal interpretations.

### Vertical interpretation from behind viewpoint

Participants made higher interpretations for rightward points compared to leftward ones. While azimuth had no effect when pointing downwards, vertical interpretations climbed continuously from left- to rightward arm orientations for points that are parallel to the floor and especially for upward gestures. Thus, arm azimuth affected vertical interpretations. Finally, interpretations were generally higher for the shortest distance compared to middle or large distance, indicating a non-linear trend in interpretations.

### Vertical interpretation from side viewpoint

Arm azimuth affected vertical interpretations in a reversed u-shape manner with highest interpretations when pointing straight ahead compared to left- and rightward points. The further the arm was oriented to the right side, the larger was the effect of elevation. Interpretations were continuously lowered over distance when pointing to the right or straight ahead compared to a slight upward tendency from 175 to 250 cm for leftward gestures. Thus, arm azimuth again affected vertical interpretations. Beyond that, the interpretations were higher when pointing to the 100 cm distant wall than to middle and large distances. At 100 cm, arm elevation increasingly influenced interpretations the more the arm was lowered while middle and large distances were not affected by arm elevation. Thus, interpretations changed non-linearly over distance.

### Short summary and interim conclusion

Results for both dimensions and viewpoints show that arm azimuth mainly determined horizontal interpretations and arm elevation vertical ones. But, more importantly, azimuth and elevation are not independently perceived from each other on either viewpoint. On the horizontal dimension, rightward gestures were interpreted more rightward the more the arm was elevated while elevation did not have much influence on leftward gestures. On the vertical dimension, elevation was perceived more extreme when the arm was oriented towards the right side. These findings imply that the individual, dimensional components of pointing gestures are understood as a configuration of different information conveyed by orientation and position of the arm. Additionally, in three out of four calculated ANOVAs, the interaction between distance and elevation (vertical dimension) or distance and azimuth (horizontal dimension) became significant indicating that observers do not make linear judgments with increasing distance. Thus, previous findings of non-linear extrapolation in two-dimensional setups are also found in three-dimensional scenarios and appears to play a role regardless of the specific viewpoint.

### Inter-individual variability within a gesture

**Fig. 6:**
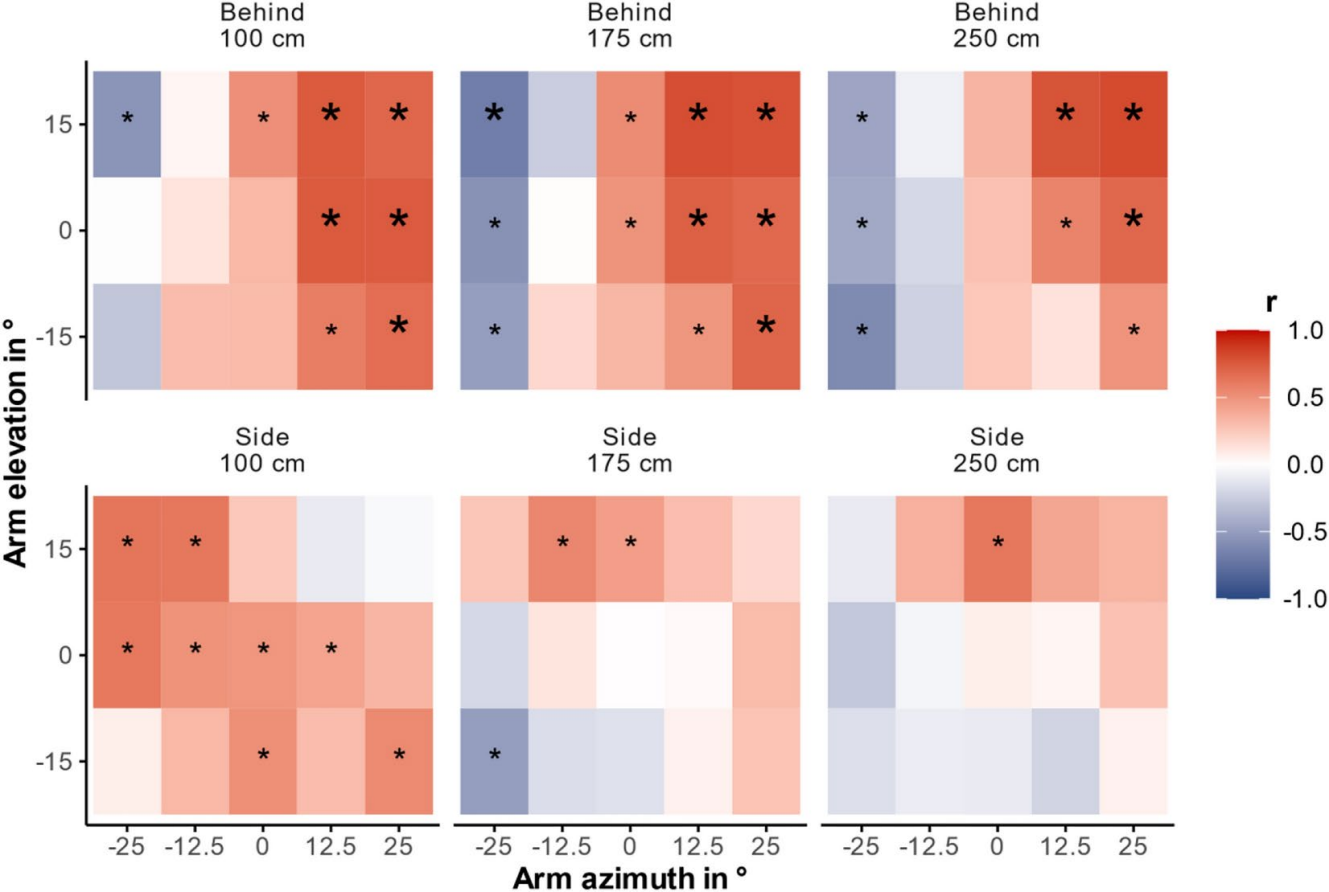
Inter-individual correlations between horizontal and vertical observer guesses. Results are separated in six facets, one for each possible combination of observer viewpoint (behind, side) and distance (100 cm, 175 cm, 250 cm). Each facet consists of 15 tiles, which correspond to all combinations of five different azimuths (−25°, −12.5°, 0°, 12.5°, 25°) and three different elevations (15°, 0°, −15°), thus all 15 different gestures. The tile’s color represents the correlation’s direction, while the color’s intensity depicts the strength of correlation. Asterisks indicate significant correlations between vertical and horizontal guesses. Correlations that survived Bonferroni-Holm correction are marked with greater, bold asterisks.

To address whether both dimensions interact in systematic individual biases in pointing perception, we averaged horizontal and vertical guesses of each factor level combination (arm azimuth (5), arm elevation (3), distance (3), observer viewpoint (2)) separated for each participant. Subsequently, we calculated correlations between the averaged guesses of both dimensions for each cell across participants. To face multiple testing problems, we adjusted the alpha level with Bonferroni-Holm correction separately for all 45 tests involved for each observer viewpoint. Figure 6 shows inter-individual correlations for each gesture (cf. exact correlation coefficients in Supplemental Table 3). Of 27 uncorrected significant correlations for behind viewpoint (positive correlations when pointed rightward, azimuths = 12.5° and 25°, and negative ones when pointed leftward, azimuth = −25°), 14 survived the correction. For side viewpoint, originally twelve correlations reached significance. The great majority were positive ones for shortest distance when pointing straight ahead or to the left. Here, after Bonferroni-Holm alpha correction no correlation was below the corrected alpha level. Finally, we tested with a binomial test separated for each viewpoint whether the number of significant correlations was above chance level (5%). This test indicated for behind viewpoint that 60% (uncorrected) significant correlations were clearly higher than the expected 5%, *p* < 0.001 (1-sided). For side viewpoint, 27% of the correlations reached (uncorrected) significance, which in turn is higher than expected by chance, *p* < 0.001 (1-sided). Therefore, systematic correlations between dimensions exists in the interpretations on inter-individual within-gesture level, both when observers standing behind the pointer and when being at his side (although it is difficult to pinpoint significant cells for this position).

On the inter-individual level, our data suggest that both dimensions are correlated instead of being processed independently from each other, favoring a configural approach. Observers who interpreted points further to the left perceived those gestures also more downward, especially when the pointing gesture was straight ahead or to the right side.

### Intra-individual variability within a gesture

**Fig. 7:**
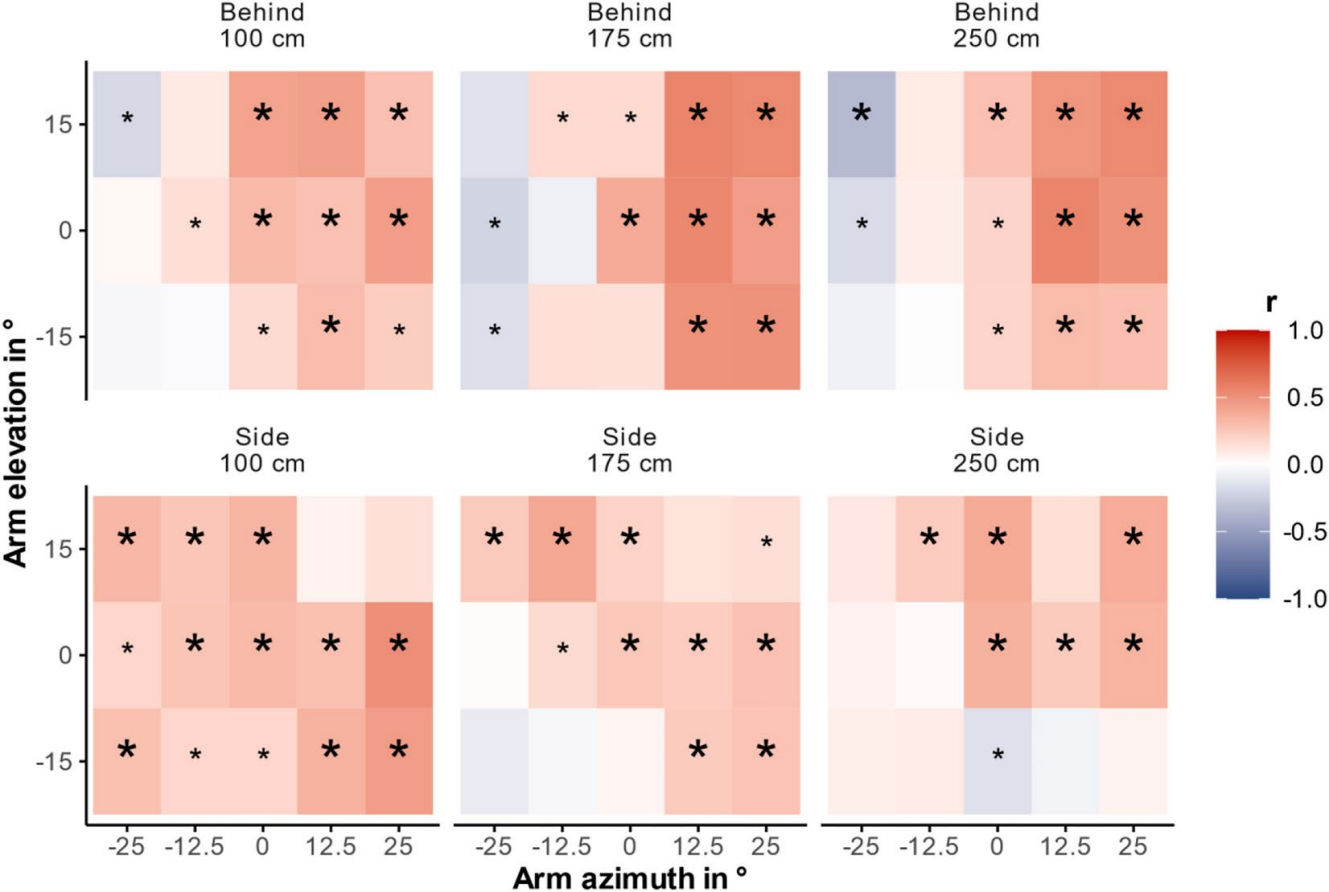
Intra-individual correlations between horizontal and vertical observer guesses. Results are separated in six facets, one for each possible combination of observer viewpoint (behind, side) and distance (100 cm, 175 cm, 250 cm). Each facet consists of 15 tiles, which correspond to all combinations of five different azimuths (−25°, −12.5°, 0°, 12.5°, 25°) and three different elevations (15°, 0°, −15°), thus all 15 different gestures. The tile’s color represents the correlation’s direction, while the color’s intensity depicts the strength of correlation. Asterisks indicate significant correlations between vertical and horizontal guesses. Correlations that survived Bonferroni-Holm correction are marked with greater, bold asterisks.

Observers provide multiple interpretations for each gesture. We calculated repeated measures correlations (rmcorr package, version 0.5.4, for R, version 4.2.3)^20,21^ to analyze a potential intra-individual relationship between vertical and horizontal interpretations of a single gesture. That is, we fit a regression line for each participant based on their individual judgments. While the regressions’ slopes are identical for all participants, their intercepts are participant depending. Therefore, separated correlations were calculated for each cell (90; combinations of arm azimuth (5), arm elevation (3), distance (3), observer viewpoint (2)). Figure 7 shows the intra-individual repeated measures correlations (cf. exact correlation coefficients in Supplementary Table 4). We applied the Bonferroni-Holm correction for multiple testing, separated for both observer viewpoints. Thus, the total number of tests was 45 for each viewpoint. For the behind viewpoint, 22 out of 33 significant correlations survived the correction. For the side viewpoint, 24 of initially 30 significant correlations survived the correction. Generally, significant correlations were mostly positive, indicating that a more leftward interpretation was also seen more downward. At the behind viewpoint, correlations were mainly significant when the pointer pointed straight ahead or to the right side, while correlations rarely reached significance after Bonferroni-Holm-correction when pointed towards the left side. At the side viewpoint, predominantly correlations on the left-/upward to right-/downward axis survived the alpha correction. Moreover, correlations were overall more positive and more frequently significant for the 100 cm distance than for the other distances. To complement the alpha correction and to check whether the number of significant correlations was above chance (5%), we again calculated binomial tests for each observer viewpoint separately. They indicated that 73% of correlations reached significance according for the behind viewpoint and 67% for the side viewpoint. Both percentages clearly exceed the percentage of significant effects that would have been expected if no actual correlation existed (5%, *p* < 0.001, 1-sided).

In sum, the data indicate a considerable structure of the interpretation variability on the intra-individual and intra-gestural level, suggesting that the vertical and the horizontal component of a pointing gesture are processed in a configural manner. In our experiment, a relatively leftward interpretation was usually also more downward.

## Discussion

A pointing gesture to a distal referent conveys information about its vertical and horizontal object position relative to the pointer. We examined how these two components interact on three levels of pointing perception and found interactions on each level. First, on between-gestures level, judgments were always influenced by both dimensions—no matter whether the observer stood behind or to the side of the pointer. More interestingly, the strength and direction of the interaction was modulated by the gesture itself. Depending on whether the pointer was pointing to the right or left, for example, arm azimuth had no influence on vertical judgments, or resulted in more extreme interpretations. Generally, vertical judgments were stronger affected by arm azimuth than horizontal ones by arm elevation. Second, the analysis of inter-individual variability on within-gesture level also revealed, that both dimensions were interwoven when considering individual biases in pointing perception. The results indicate that the further to the right the gesture was seen, the higher the elevation was estimated. However, when gestures indicated a pointed-at location at the left side, gestures were perceived less extreme to the left side but at the same time further upward. Third, for intra-individual variability on the within-gesture level, gestures were likewise perceived mainly as further upward, when simultaneous being perceived further rightward. Thus, pointing gestures are processed in a configural way on each examined level.

The results add another facet to our understanding of the complex process of spatial interpretation of pointing gestures and illustrate once again the intricacy of pointing perception. Vertical and horizontal dimension of the pointing gesture are intertwined on all examined levels. Even though we constantly found that points, which were judged rather rightward, were also judged rather upward, the direction of this correlation might switch when observers hold another perspective, e.g., when standing at the pointer’s left side. Therefore, the fact that these correlations exist is of greater importance than their specific sign. Thus, to describe a pointing gesture and its interpretation accordingly, the frequently used geometrical approach with an isolated view on each dimension of the gesture should be extended as it leaves out significant aspects of the judgments. In the following, we discuss a possible explanation for the cross-dimensional interaction, distance-dependent effects in our results, and possible limitations of this study.

### Pattern of cross-dimensional effects

The analyses of cross-dimensional effects on both within-gesture levels and viewpoints revealed a similar pattern. Albeit no formal statistical analysis has been conducted, we here want to offer an explanation for the distribution of cross-dimensional effects. When the gesture was oriented towards the lower left, we found only little or no correlations between both horizontal and vertical interpretations. This pattern is most prominent for the intra-individual correlations at the behind viewpoint but also emerges for the inter-individual correlations and the side viewpoint. When the arm was pointing down- and leftward, the gesture was highly aligned with the gaze of the observer. Thus, the observer looked down the arm, presumly finding the finger position whithin their visual field highly plausible to indicate the pointed-at position (position cue)^9^. By contrast, a cross-dimensional effect was often found when the gesture was orientated up- or rightwards. When the angle between the pointer’s shoulder and his index finger and the observer’s eyes became wider, observers tend to extrapolate from the fingertip in these cases (direction cue)^9^. This pattern is present but weaker for the side viewpoint because the arm is always seen from the side and the additional effect of the arm orientation is small in comparison.

This pattern suggests that cross-dimensional effects may be mainly attributed to the extrapolation from the pointer’s fingertip, that is, the use of the direction cue. Whether its higher perceptual uncertainty, compared to the position cue^19^, opens the gateway for other factors to play out or whether the extrapolation process itself introduces the cross-dimensional effects, for examples due to uncertainty in the perception of the arm orientation, is an open question.

### Distance

Regarding distance, two findings were striking. First, pointing gestures were extrapolated non-linearly over distance. As observers’ responses were measured in angles extending from pointing finger, averaged values of different distances should be superimposed in Figs. 4 and 5 in case observers extrapolated judgments linearly and began the extrapolation at pointer’s fingertip. However, the charts reveal that this was not the case, illustrating once again that observers’ estimations were non-linearly distorted over distance^6,22^. This non-linearity was mainly apparent between the shortest distance and the wider distances at both viewpoints. Between 175 and 250 cm conditions, observers showed a judgment pattern indicating mostly linear extrapolation in the great majority.

Previously, it has been found that vertical interpretations of pointing gestures were biased the more to horizonal axes, the more distant the pointer was from the referent area^6^. As we reported interpretations as angles of the vector from fingertip to guess, we would have expected that the effect of arm azimuth or elevation on horizontal and vertical interpretations, respectively, decreases with distance. While such a pattern increasingly appeared for the horizontal component of behind viewpoint with increasing arm azimuth, a reversed pattern emerged for the vertical dimension from side viewpoint. Therefore, the data suggest that previous findings of this central tendency effect does not generalize to all situations.

However, in our study no effect of distance was found for horizontal judgments made from side viewpoint indicating neither a fanning out of judgments nor a central tendency. On closer inspection, a faint central tendency for leftward gestures between 175 and 250 cm distances occurred while at least a corresponding tendency appeared for rightward arm orientations. Although often found in previous studies, it is still not clear why judgements are generally biased towards a sagittal axis. By contrast, we now found some situations in which judgments grow more extreme over distance. Identifying the mechanism that guide non-linear interpretations in general and moreover explains what factors determine the direction of this bias—growing more extreme over distance or showing a central tendency—will be addressed in future studies. Notwithstanding, with this experiment we could show that non-linear judgments are not limited to vertical dimension but also occur for horizontal pointing interpretation.

The second striking result concerning distance is that observers’ interpretations become increasingly linear from shortest to longest distance when standing behind the pointer’s shoulder. At first glance, it might appear as if it would be somewhat easier for observers from behind viewpoint to extend the gesture. While this might be true for interpretations from 175 to 250 cm where extrapolations were rather linear, judgments from 100 to 175 cm were pulled quite up- and rightwards, peculiarly for points to the upper, right corner. This pattern visually resembles the one for interpretations made from sideward observer viewpoint.

As the gap between index fingertip and wall was only about 24 cm wide in the 100 cm condition, results might be explained by observers perceiving the pointer as touching the wall when standing behind the pointer’s shoulder. However, results indicate that observers consider the gap in their interpretations (see Supplement 1). Furthermore, this pattern might result from a considerable reduction of depth cues since the wall covered almost the entire visual field apart from a narrow strip on the left at the beginning of each trial at side viewpoint in 100 cm condition. At the behind viewpoint, even the whole visual field in the HMD was covered by the wall. Thus, we checked whether observers might have under- or overestimated the distance and consequently marked other pointed-at locations as intended. However, this was not the case neither at behind viewpoint nor at side viewpoint (see Supplement 2). This finding is supported by Surber and colleagues who found that small distances just beyond the reachable space in virtual reality are estimated quite accurately^8^. In sum, the non-linearity of pointing interpretations neither results from participants perceiving the pointer as touching the wall nor from participants misperceiving the distance of the 100 cm wall.

### Limitations

We conducted our experiment in virtual reality with an artificial, static pointer whose gestures were modelled after what is sometimes called the canonical form of pointing^1^—index finger point with extended straight arm—and which is typically observed in experiments and the wild^1,2,6,23,24^. However, as we did not rely on motion-capture data, the nuances of natural human pointing (such as movements or the exact hand posture) may not be properly reflected in our experiment. Nevertheless, we are convinced that our findings transfer to real-world situations. For example, Krause and Herbort^9^ conducted an experiment in which a virtual person pointed, and participants were asked to mark the perceived pointed-at locations. In a follow-up experiment, they transferred task and design into a real-world set-up with a human pointer. The human pointer was asked to point at the exact locations that were also indicated by the virtual pointer from the first experiment. The data showed that the interpretation pattern was very similar between VR and real-world environment. As the current experiment resembled that of Krause and Herbort^9^ with respect to the virtual pointer, gesture production, and spatial layout, our results likely also transfer to the (unmediated) interpretation of human pointing. Furthermore, Wong and Gutwin^22^ directly compared both environments regarding judgment accuracy over distance. Although they found a significant effect of environment, accuracy differences in pointing interpretation were nominal rather small (1.4° over 300 cm and 0.33° over 600 cm), with errors generally diminishing over distance and interpretations made in real-world environment being only slightly more accurate^22^. Finally, pointer-observer misunderstandings of naïve participants could be predicted based on interpretations of computer-generated pointing gestures^6^. Thus, although virtual pointing gestures may not capture all nuances of real-world pointing, their interpretations do not differ qualitatively from natural pointing gestures.

### Summary

The experiment revealed that the perception of pointing gestures is not only affected by factors like distance or observer viewpoint but also by the interaction of vertical and horizontal aspects of the pointing gesture. This reciprocal influence of the arm azimuth on vertical judgments and likewise of the arm elevation on horizontal judgments showed up on all three investigated levels. In more detail: First, gesture perception is more complex than previously thought, since horizontally identical arm orientations are assessed differently depending on the vertical information the arm is conveying. Likewise, this also applied for the influence of horizontal pointing component on vertical judgments. Second, observers show rather similar systematic interactions in vertical and horizontal interpretations of a pointing gesture, supporting the idea of configural processing. Generally said, points were perceived relatively higher when seen further rightwards. Third, dimensions also interacted within a single pointing episode on intra-individual level. Fourth, observers showed once again non-linear extrapolation patterns within their interpretation on vertical but also on horizontal dimension. Surprisingly and contrary to previous studies and horizontal judgments in the actual study, vertical guesses rather clustered in principle not around a sagittal axis but fanned out from short to the longer distances.

## Supplementary Information

Supplementary Information.

## Data Availability

Data and analysis scripts are available at Open Science Framework (https://osf.io/snd7r/).
