# Supplementary material for: Perception of pointing gestures in 3D space
Source: Sci Rep. 2024 Nov 11;14:27595. doi: 10.1038/s41598-024-78129-4 (PMC11554782; doi:10.1038/s41598-024-78129-4)
Supplement: Supplementary file 1 — Supplementary Information. [file 41598_2024_78129_MOESM1_ESM.docx]

# Supplemental Material

## Supplemental Table 1

*Results of ANOVA for Horizontal Dimension*

| Effect | *F* | *df* | *p* | $\eta_{p}^{2}$ | *ε* |
| --- | --- | --- | --- | --- | --- |
| Observer Viewpoint | **18.77** | **1, 21** | **<.001** | **.47** | **-** |
| Arm Azimuth | **994.90** | **4,84** | **<.001** | **.98** | **.37** |
| Arm Elevation | **11.93** | **2,42** | **<.001** | **.36** | **.86** |
| Distance | 0.39 | 2,42 | .553 | .02 | .54 |
| Viewpoint x Azimuth | **48.38** | **4,84** | **<.001** | **.70** | **.36** |
| Viewpoint x Elevation | **10.02** | **2,42** | **<.001** | **.32** | **.94** |
| Viewpoint x Distance | 1.83 | 2,42 | .189 | .08 | .55 |
| Azimuth x Elevation | **26.30** | **8,168** | **<.001** | **.56** | **.60** |
| Azimuth x Distance | **6.73** | **8,168** | **<.001** | **.24** | **.35** |
| Elevation x Distance | **3.72** | **4,84** | **.018** | **.15** | **.71** |
| Viewpoint x Azimuth x Elevation | **10.08** | **8,168** | **<.001** | **.32** | **.62** |
| Viewpoint x Azimuth x Distance | **4.96** | **8,168** | **.006** | **.19** | **.32** |
| Viewpoint x Elevation x Distance | **3.94** | **4,84** | **.012** | **.16** | **.76** |
| Azimuth x Elevation x Distance | **3.51** | **16,336** | **.002** | **.14** | **.41** |
| Viewpoint x Azimuth x Elevation x Distance | **3.36** | **16,336** | **.003** | **.14** | **.43** |

*Note*. Significant main effects and interactions are printed in bold.

## Supplemental Table 2

*Results of ANOVA for Vertical Dimension*

| Effect | *F* | *df* | *p* | $\eta_{p}^{2}$ | *ε* |
| --- | --- | --- | --- | --- | --- |
| Observer Viewpoint | **107.59** | **1,21** | **<.001** | **.84** | **-** |
| Arm Azimuth | **14.89** | **4,84** | **<.001** | **.42** | **.44** |
| Arm Elevation | **1429.12** | **2,42** | **<.001** | **.99** | **.54** |
| Distance | **28.90** | **2,42** | **<.001** | **.58** | **.77** |
| Viewpoint x Azimuth | **15.54** | **4,84** | **<.001** | **.43** | **.61** |
| Viewpoint x Elevation | 1.81 | 2,42 | .190 | .08 | .63 |
| Viewpoint x Distance | **7.11** | **2,42** | **.005** | **.25** | **.79** |
| Azimuth x Elevation | **74.27** | **8,168** | **<.001** | **.78** | **.58** |
| Azimuth x Distance | **3.30** | **8,168** | **.007** | **.14** | **.66** |
| Elevation x Distance | **22.20** | **4,84** | **<.001** | **.51** | **.71** |
| Viewpoint x Azimuth x Elevation | **19.24** | **8,168** | **<.001** | **.48** | **.50** |
| Viewpoint x Azimuth x Distance | **2.48** | **8,168** | **.036** | **.11** | **.63** |
| Viewpoint x Elevation x Distance | **17.58** | **4,84** | **<.001** | **.46** | **.69** |
| Azimuth x Elevation x Distance | 1.82 | 16,336 | .096 | .08 | .40 |
| Viewpoint x Azimuth x Elevation x Distance | 0.63 | 16,336 | .723 | .03 | .42 |

*Note*. Significant main effects and interactions are printed in bold.

## Supplemental Table 3

*Correlation Coefficients for Inter-Individual and Intra-Gestural Analyses*

|  |  |  | Right Behind | | |  | Right Side | | | | |  |
| --- | --- | --- | --- | --- | --- | --- | --- | --- | --- | --- | --- | --- |
| Azimuth | Elevation |  | 100 cm | 175 cm | 250 cm |  | 100 cm | 175 cm | | 250 cm | |  |
| -25° | 15° |  | -0.554** | -0.681** | -0.464* |  | 0.631** | | 0.266 | | -0.101 | |
|  | 0° |  | -0.011 | -0.570** | -0.442* |  | 0.617** | | -0.196 | | -0.280 | |
|  | -15° |  | -0.289 | -0.504* | -0.609** |  | 0.073 | | -0.505* | | -0.159 | |
| -12.5° | 15° |  | 0.040 | -0.253 | -0.074 |  | 0.624** | | 0.555** | | 0.367 | |
|  | 0° |  | 0.126 | 0.012 | -0.199 |  | 0.502* | | 0.122 | | -0.052 | |
|  | -15° |  | 0.307 | 0.179 | -0.232 |  | 0.332 | | -0.169 | | -0.089 | |
| 0° | 15° |  | 0.512* | 0.530* | 0.347 |  | 0.253 | | 0.450* | | 0.623** | |
|  | 0° |  | 0.332 | 0.497* | 0.282 |  | 0.486* | | 0.008 | | 0.074 | |
|  | -15° |  | 0.313 | 0.339 | 0.260 |  | 0.513* | | -0.150 | | -0.104 | |
| 12.5° | 15° |  | 0.751** | 0.801** | 0.777** |  | -0.103 | | 0.303 | | 0.410 | |
|  | 0° |  | 0.750** | 0.724** | 0.570** |  | 0.423* | | 0.021 | | 0.040 | |
|  | -15° |  | 0.594** | 0.483* | 0.131 |  | 0.310 | | 0.063 | | -0.217 | |
| 25° | 15° |  | 0.699** | 0.786** | 0.816** |  | -0.032 | | 0.180 | | 0.351 | |
|  | 0° |  | 0.749** | 0.689** | 0.696** |  | 0.344 | | 0.315 | | 0.280 | |
|  | -15° |  | 0.673** | 0.703** | 0.495* |  | 0.542** | | 0.273 | | 0.064 | |

*Note.* Correlations were generated by averaging horizontal and vertical estimations for all factor-level combinations of each participant. Guesses for both dimensions were correlated for each cell across participants. * *p* < .05, ** *p* < .01

## Supplemental Table 4

*Correlation Coefficients for Intra-Individual and Intra-Gestural repeated measures analyses*

|  |  |  | Right Behind | | |  | Right Side | | | | |  |
| --- | --- | --- | --- | --- | --- | --- | --- | --- | --- | --- | --- | --- |
| Azimuth | Elevation |  | 100 cm | 175 cm | 250 cm |  | 100 cm | 175 cm | | 250 cm | |  |
| -25° | 15° |  | -0.191* | -0.145 | -0.349** |  | 0.328** | | 0.246** | | 0.104 | |
|  | 0° |  | 0.035 | -0.218** | -0.179* |  | 0.188** | | 0.019 | | 0.059 | |
|  | -15° |  | -0.039 | -0.162* | -0.068 |  | 0.293** | | -0.101 | | 0.073 | |
| -12.5° | 15° |  | 0.102 | 0.168* | 0.098 |  | 0.265** | | 0.402** | | 0.233** | |
|  | 0° |  | 0.146* | -0.077 | 0.082 |  | 0.270** | | 0.168* | | 0.023 | |
|  | -15° |  | -0.023 | 0.140 | -0.010 |  | 0.190** | | -0.039 | | 0.086 | |
| 0° | 15° |  | 0.427** | 0.172* | 0.287** |  | 0.344** | | 0.207** | | 0.390** | |
|  | 0° |  | 0.319** | 0.388** | 0.200** |  | 0.318** | | 0.256** | | 0.358** | |
|  | -15° |  | 0.166* | 0.138 | 0.194** |  | 0.189* | | 0.047 | | -0.157* | |
| 12.5° | 15° |  | 0.438** | 0.561** | 0.488** |  | 0.060 | | 0.126 | | 0.145 | |
|  | 0° |  | 0.285** | 0.549** | 0.560** |  | 0.289** | | 0.232** | | 0.239** | |
|  | -15° |  | 0.310** | 0.501** | 0.317** |  | 0.354** | | 0.239** | | -0.060 | |
| 25° | 15° |  | 0.291** | 0.542** | 0.545** |  | 0.137 | | 0.148* | | 0.386** | |
|  | 0° |  | 0.448** | 0.451** | 0.504** |  | 0.512** | | 0.280** | | 0.351** | |
|  | -15° |  | 0.230** | 0.504** | 0.297** |  | 0.453** | | 0.278** | | 0.055 | |

*Note.* Horizontal and vertical guesses were correlated within each factor combination with repeated measures correlation. * *p* < .05, ** *p* < .01

## Supplemental Text 1

We checked whether observers might have perceived gestures in the 100 cm condition as touching the wall when standing behind the pointer’s shoulder. Therefore, we spanned a vector from observer’s cyclopean eye over the pointer’s fingertip towards the wall to calculate the location where on the wall the fingertip was seen (Supplemental Fig. 1). If the gesture had been seen as touch, one could have expected that observers mark the calculated location underneath the fingertip or at least a location adjacent to it. Instead, locations above – and mostly to the right of – the fingertip position in their visual field were clicked on. That demonstrates again that observers at least slightly extrapolate their judgments when sharing the same perspective with the pointer albeit to a lesser extent compared to side viewpoints. There at side viewpoint, direction cue is mainly determining the estimation. We found the small gap between pointer and wall was noticed and taken into account in the observers’ interpretations accordingly.

**Supplemental Figure 1**

*Judgments made from Behind Viewpoint and Locations Covered by the Pointer’s Fingertip for each Gesture*


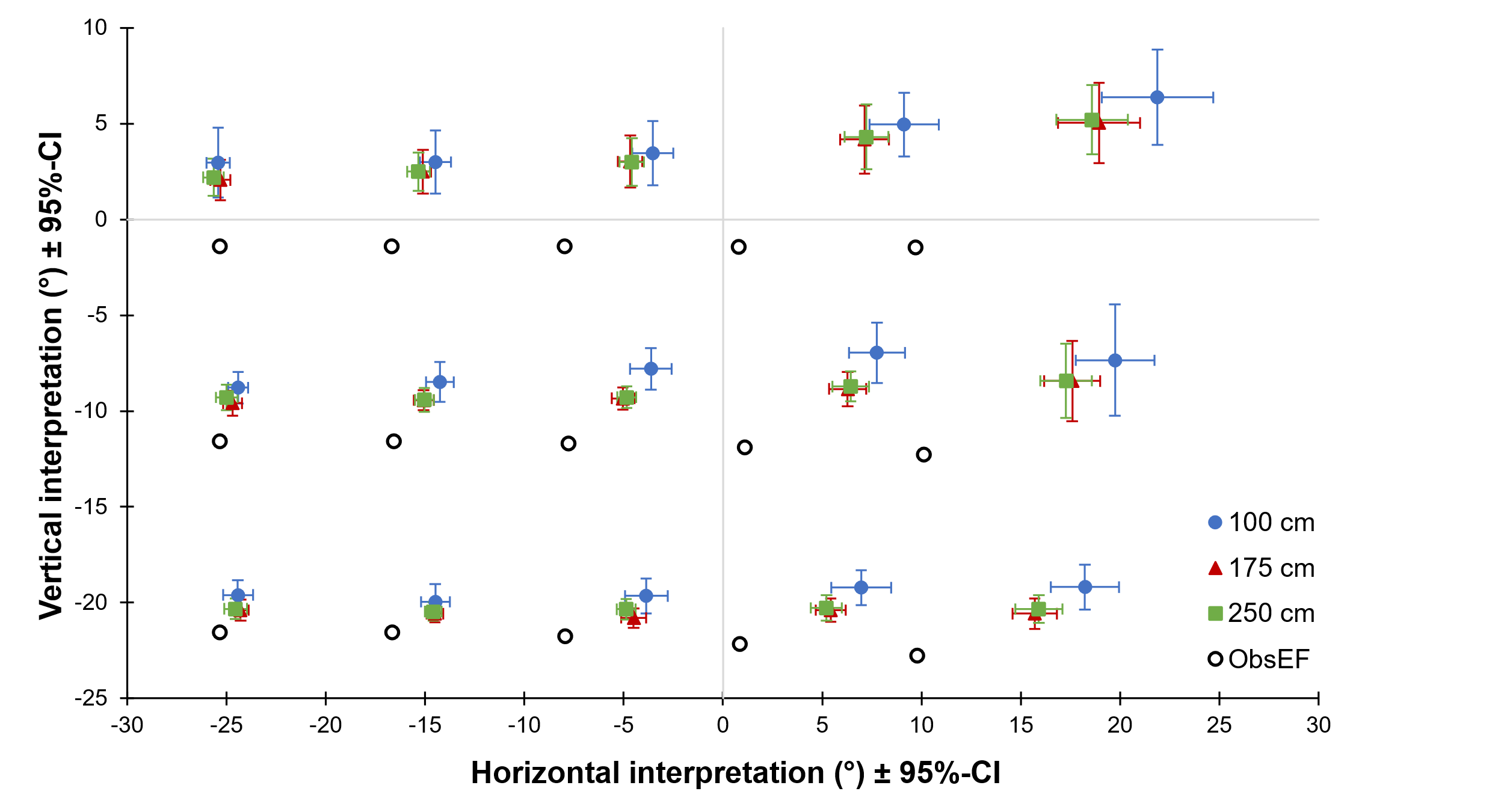


*Note*. Vertical and horizontal judgments made from behind viewpoint for each gesture and distance level. Black circles mark the theoretical locations where observers saw the pointer’s fingertip on the wall in their visual field (the intersection of the vector starting in the observer’s cyclopean eye to the pointer’s finger towards the wall). The real location might deviate to some point as participants were allowed to move their heads up to 15 cm around the predefined observer viewpoints.

## Supplemental Text 2

We checked whether observers might have under- or overestimated the distance to the wall in the 100 cm condition. Thus, we post-hoc calculated the vertical and horizontal interpretation that would have resulted when the cursor stayed at the same position in the participants’ visual field, but the wall would have been at a distance between 80 cm and 120 cm. Subsequently, we determined the averaged distance between the computed “virtual” guess (assuming a misperception of the 100 cm distance) and the actual guesses for the two larger distances (175 cm and 250 cm) in angular degree. If the recomputed guesses at the nominal 100 cm distance would move closer to the guesses from the 175 cm and 250 cm distance conditions for a hypothetical perceived distance, this would indicate that participants may have extrapolated linearly but have misperceived the 100 cm distance. However, this was not the case. The closest match resulted when it was assumed that participants perceived the 100 cm wall as being 99 cm distant, which is arguably very close to the nominal value.
